# Agriculture creates subtle genetic structure among migratory and nonmigratory populations of burrowing owls throughout North America

**DOI:** 10.1002/ece3.6725

**Published:** 2020-09-17

**Authors:** Alberto Macías‐Duarte, Courtney J. Conway, Melanie Culver

**Affiliations:** ^1^ Arizona Cooperative Fish and Wildlife Research Unit School of Natural Resources and the Environment University of Arizona Tucson AZ USA; ^2^ Idaho Cooperative Fish & Wildlife Research Unit US Geological Survey University of Idaho Moscow ID USA; ^3^ Arizona Cooperative Fish and Wildlife Research Unit US Geological Survey University of Arizona Tucson AZ USA

**Keywords:** *Athene cunicularia hypugaea*, dispersal, DNA microsatellites, gene flow, genetic differentiation, irrigated agriculture, migration, population genetics

## Abstract

Population structure across a species distribution primarily reflects historical, ecological, and evolutionary processes. However, large‐scale contemporaneous changes in land use have the potential to create changes in habitat quality and thereby cause changes in gene flow, population structure, and distributions. As such, land‐use changes in one portion of a species range may explain declines in other portions of their range. For example, many burrowing owl populations have declined or become extirpated near the northern edge of the species' breeding distribution during the second half of the 20th century. In the same period, large extensions of thornscrub were converted to irrigated agriculture in northwestern Mexico. These irrigated areas may now support the highest densities of burrowing owls in North America. We tested the hypothesis that burrowing owls that colonized this recently created owl habitat in northwestern Mexico originated from declining migratory populations from the northern portion of the species' range (migration‐driven breeding dispersal whereby long‐distance migrants from Canada and the United States became year‐round residents in the newly created irrigated agriculture areas in Mexico). We used 10 novel microsatellite markers to genotype 1,560 owls from 36 study locations in Canada, Mexico, and the United States. We found that burrowing owl populations are practically panmictic throughout the entire North American breeding range. However, an analysis of molecular variance provided some evidence that burrowing owl populations in northwestern Mexico and Canada together are more genetically differentiated from the rest of the populations in the breeding range, lending some support to our migration‐driven breeding dispersal hypothesis. We found evidence of subtle genetic differentiation associated with irrigated agricultural areas in southern Sonora and Sinaloa in northwestern Mexico. Our results suggest that land use can produce location‐specific population dynamics leading to subtle genetic structure even in the absence of dispersal barriers.

## Graphical abstract

Migration‐driven breeding dispersal in the burrowing owl, whereby long‐distance migrants from Canada and the United States became year‐round residents in the newly created irrigated agriculture areas in northwestern Mexico, may explain the species' population decline in northern latitudes. In support of this hypothesis, (a) owl populations in northwestern Mexico and Canada together are more genetically differentiated from the rest of the populations, and (b) there is a subtle genetic differentiation associated with irrigated agricultural areas in northwestern Mexico.

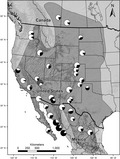

## 1 INTRODUCTION

Understanding ecological and evolutionary dynamics of a species at the edges of its distribution can help unveil the mechanisms that limit abundance throughout a species' entire geographic range (Holt & Keitt, 2005). In this regard, ecological theory and empirical evidence support the idea that species tend to be less abundant and more prone to local population extinction at the periphery of their geographic ranges (Gaston, 2003). Populations at the edge of a species' distribution may be maintained by dispersal and recolonization from interior populations (Curnutt, Pimm, & Maurer, 1996). This scenario whereby populations on the periphery are repeatedly “rescued” (Brown & Kodric‐Brown, 1977) by interior populations may be particularly important for species of conservation concern. Understanding the processes by which peripheral populations are maintained in those species is important for designing effective recovery efforts. For example, populations of the western burrowing owl (*Athene cunicularia hypugaea*) have been extirpated from some areas and are rare and declining in other areas near the northern edge of their breeding distribution (Clayton & Schmutz, 1999; Macías‐Duarte & Conway, 2015; Skeel, Keith, & Palaschuk, 2001; Wellicome & Holroyd, 2001). The breeding range of the western burrowing owl (burrowing owl hereafter) historically comprised semiarid grasslands from southern Canada to central Mexico (Poulin, Todd, Haug, Millsap, & Martell, 2020). Hypotheses to explain population declines in the northern portion of their range include local mechanisms such as conversion of grassland to dryland farming in the northern Great Plains, extirpation of black‐tailed prairie dogs, toxicological effects of pesticides, collisions with vehicles, and annual dispersal (Clayton & Schmutz, 1999; Desmond, Savidge, & Eskridge, 2000; Duxbury, 2004; Klute et al., 2003; Poulin et al., 2020). All these hypotheses seem insufficient to explain the extent of burrowing owl population declines observed in the northern portion of their breeding range because many areas with seemingly suitable habitat remain unoccupied. Nevertheless, the highest breeding densities occur in the southern portion of the burrowing owl's breeding range (in Imperial Valley, California; DeSante, Ruhlen, & Rosenberg, 2004; Rosenberg & Haley, 2004; Sauer et al., 2017). In addition, densities of breeding burrowing owls in the coastal plains of Sonora and Sinaloa may be just as high as those in southeastern California (Macías‐Duarte, 2011). These high densities of burrowing owls in the southern portions of the species' range are all in arid desert areas that have been converted to irrigated agriculture. High densities of breeding burrowing owls in this portion of their range is a recent phenomenon; more than 1.5 million hectares of coastal thornscrub and tropical dry forest in Sonora and Sinaloa were converted to irrigated farmland in the last 60 years (Anonymous, 1994; Rohwer, Grason, & Navarro‐Sigüenza, 2015). This redistribution of burrowing owls (the breeding range contracting in the north and expanding in the south) poses interesting questions about the mechanisms that shape and maintain the geographic range of the species especially given that many other birds in North America are showing opposite trends (ranges shifting northward) in response to climate change (La Sorte & Thompson, 2007). In this paper, we propose and test the hypothesis that the contraction at the northern periphery of the burrowing owl's range and the expansion in the southern portion of their range may be directly related.

Most breeding populations of burrowing owls in North America exhibit partial migration (where some individuals migrate and some do not), but northern populations in the Great Plains are 100% migratory (Poulin et al., 2020). James (1992) speculated that burrowing owls have a leap‐frog migration pattern (negative correlation between breeding latitude and wintering latitude across populations). In addition, most burrowing owls that breed in the northern portion of the breeding range appear to spend their winters in southern Texas and central Mexico (Duxbury, 2004; Holroyd, Trefry, & Duxbury, 2010). We tested the hypothesis that burrowing owls that once migrated annually from northern portions of their breeding range to central Mexico became resident breeders in these newly created irrigated agricultural areas, contributing to both population declines in the north and population increases in the south. Birds breeding within what was formerly their wintering grounds (migrants becoming year‐round residents) has been contemporarily observed in at least 3 other species (Sutherland, 1998). However, numerous phylogenetic analyses infer that these migratory drop‐offs have been common through the evolutionary histories of migratory birds and are drivers of diversification and speciation (e.g., Gómez‐Bahamón et al., 2020; Rolland, Jiguet, Jønsson, Condamine, & Morlon, 2014; Voelker & Light, 2011; Winger, Barker, & Ree, 2014).

Testing this hypothesis requires inferring patterns of breeding dispersal (movement between two breeding attempts; Greenwood & Harvey, 1982) among populations throughout the burrowing owl breeding range. We used genetic markers to infer the patterns of gene flow produced by breeding dispersal by measuring genetic differentiation among migratory and nonmigratory populations throughout North America. We tested 3 predictions of our hypothesis that infer patterns of genetic variation produced by gene flow from northern migratory (declining) populations to southern populations within irrigated agricultural areas. First, our hypothesis predicts that genetic differentiation between a northern migratory population and a southern agricultural population will be lower than the expected genetic differentiation predicted by the geographic distance between the 2 populations. This prediction assumes an isolation‐by‐distance pattern (Wright, 1943), where populations further apart geographically are more genetically differentiated (however, subtle) than populations closer to each other due to differences in frequency of dispersal. Second, our hypothesis predicts that northern migratory populations and southern agricultural populations together are genetically similar enough to be differentiated from the rest of the breeding populations within the burrowing owl breeding range. This prediction can be tested via a significance test of the two‐group classification of burrowing owl populations mentioned above to explain overall genetic variation. We can use an assignment test to test a third prediction. Assignment tests use individual genotypes to estimate the probability of membership of each individual genotype to predefined clusters of individuals. In this regard, our hypothesis predicts that southern agricultural populations will have more individual owls with probabilities of membership similar to those found in individuals from northern migratory populations (in areas where owls are declining) compared to the nonagricultural populations in the southern part of the species' range. We used DNA samples from owls throughout their North American breeding range to test these 3 predictions.

## 2 METHODS

### 2.1 Study area

**Figure 1:**
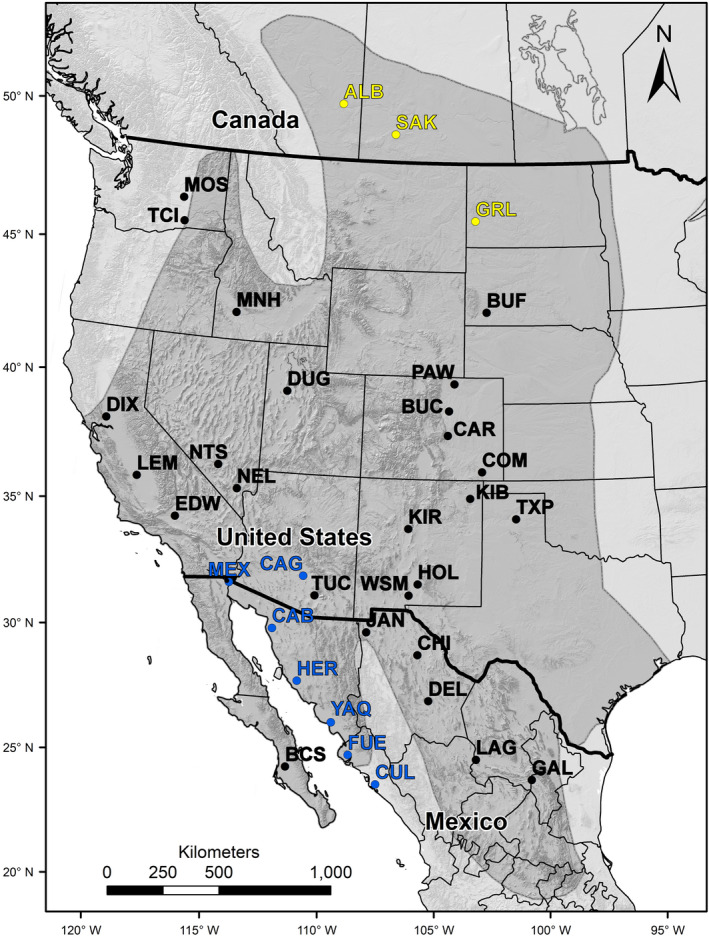
Burrowing owl study locations in Canada, Mexico, and the United States. Acronyms for study locations are listed in Table 1. Yellow labels denote northern declining migratory populations and blue labels denote southern agricultural populations. The gray area denotes the breeding distribution of the burrowing owl(after Poulin et al., 2020)

We obtained DNA samples from 1,560 breeding burrowing owls from 36 locations (‘study locations’ hereafter) in Canada, Mexico, and the United States (Figure 1, Table 1). To test our predictions, we grouped the 36 study locations into 3 categories: Agricultural areas in the southern portion of the species' range, areas in the northern portion of the species' range where migratory populations are declining, and all other study locations. Seven of our study locations were located in irrigated agricultural areas of northwestern Mexico and southern Arizona (“southern agricultural study locations” hereafter). These study locations were Casa Grande (CAG), Mexicali Valley (MEX), Caborca (CAB), Hermosillo (HER), Yaqui‐Mayo Valley (YAQ), Rio Fuerte Valley (FUE), and Culiacan (CUL). Some population declines have been documented throughout the breeding range of the burrowing owl, but systematic regional declines have been most evident in Alberta, Saskatchewan, North Dakota, and South Dakota, where the species is close to extirpation (owls have been extirpated from Manitoba and British Columbia). Therefore, we only defined Alberta (ALB), Saskatchewan (SAK), and Grand River‐Little Missouri National Grasslands (GRL) as northern study locations with declining migratory breeding populations (“northern study locations” hereafter) (Table 1).

**Table 1:** Numbers of individuals sampled within each of 36 burrowing owl study locations in Canada, United States, and Mexico

Study location	Acronym	Individuals genotyped
Southern Alberta, Alberta, Canada	ALB†	37
Baja California Sur, Mexico	BCS	23
Buckley Air Force Base, Colorado, USA	BUC	33
Buffalo Gap National Grassland, South Dakota, USA	BUF	54
Caborca Valley, Sonora, Mexico	CAB*	25
Casa Grande, Arizona, USA	CAG*	59
Fort Carson Army Base, Colorado, USA	CAR	23
Coyame and Ahumada, Chihuahua, Mexico	CHI	34
Comanche National Grassland, Colorado, USA	COM	40
Culiacan Valley, Sinaloa, Mexico	CUL*	63
Delicias, Chihuahua, Mexico	DEL	25
Dixon Naval Radio Transmitter Facility, California, USA	DIX	29
Dugway Air Force Base, Utah, USA	DUG	30
Edwards Air Force Base, California, USA	EDW	44
Rio Fuerte Valley, Sinaloa, Mexico	FUE*	67
Galeana, Nuevo Leon, Mexico	GAL	47
Grand River‐Little Missouri Natl. Grasslands, North Dakota	GRL†	21
Hermosillo, Sonora, Mexico	HER*	60
Holloman Air Force Base, New Mexico, USA	HOL	22
Janos, Chihuahua, Mexico	JAN	62
Kiowa ‐ Rita Blanca National Grasslands, NM, TX, USA	KIB	29
Kirtland Air Force Base, New Mexico, USA	KIR	73
La Laguna, Coahuila, Mexico	LAG	54
Naval Air Station Lemoore, California, USA	LEM	47
Mexicali Valley, Baja California, Mexico	MEX*	59
Mountain Home Air Force Base, Idaho, USA	MNH	62
Moses Lake, Washington, USA	MOS	55
Nellis Air Force Base, Nevada, USA	NEL	55
Nevada Test Site, Nevada, USA	NTS	25
Pawnee National Grassland, Colorado, USA	PAW	54
Grasslands National Park and Regina Plains, Saskatchewan	SAK†	61
Tri‐Cities, Washington, USA	TCY	54
Tucson, Arizona, USA	TUC	25
Texas Panhandle, Texas, USA	TXP	15
White Sands Missile Range, New Mexico, USA	WSM	24
Yaqui‐Mayo Valley, Sonora, Mexico	YAQ*	70

Note

Study location acronyms with (*) and (†) denote southern agricultural populations and northern declining migratory populations, respectively.

### 2.2 Sample collection

We trapped burrowing owls during the summers of 2004–2009. We trapped burrowing owls using push‐door tramps (Winchell, 1999) set at the entrance of nest burrows and bownet traps (Bub, 1991) set near nest burrows. None of the 1,560 birds that we included in our analysis were closely related (i.e., a parent and its offspring, or >1 juvenile from the same nest burrow). Our primary source of genomic DNA was blood. We obtained ~50 μl of blood through a venipuncture of the brachial vein. We also used flight and/or body feathers occasionally as a source of genomic DNA when we could not withdraw a blood sample.

### 2.3 Genotyping

We used 10 microsatellite markers developed specifically for this study (Macías‐Duarte, Conway, Vega‐Munguía, & Culver, 2010) to obtain genotypic data from our 36 study locations. We followed the manufacturer's protocols in the DNeasy Blood & Tissue Kit (Qiagen^®^) to isolate genomic DNA from <25 μl of blood. We performed PCR reactions in a 15 μl volume containing 10–50 ng genomic DNA, 1X PCR buffer (20 mM Tris–HCl pH 8.4, 50 mM KCl, Invitrogen^®^), 0.2 mM each dNTP, 0.02 μM unlabeled M13‐tailed forward primer, 0.2 μM reverse primer pig‐tailed with GTGTCTT, 0.2 μM fluorescently labeled M13 primer, 2 mM MgCl_2_, 0.4 U Taq DNA polymerase (Invitrogen^®^), and 0.02% BSA. We used 1 touchdown protocol for all loci consisting of an initial denaturation at 94°C for 4 min followed by 10 cycles at 94°C for 30 s, annealing at 60–52°C for 90 s (2°C decrease every 2 cycles), extension at 72°C for 30 s, followed by 30 cycles at 94°C for 30 s, annealing at 50°C for 30 s and 72°C for 30 s, and a final extension of 7 min at 72°C. We analyzed PCR products on an Applied Biosystems 3730 Genetic Analyzer and used an Applied Biosystems Genotyper 3.7 to score alleles. We used program Tandem (Matschiner & Salzburger, 2009) to assign integers to DNA fragment sizes. We used program Micro‐Checker (Van Oosterhout, Hutchinson, Wills, & Shipley, 2004) to identify null alleles (Chakraborty, Deandrade, Daiger, & Budowle, 1992).

### 2.4 Data analysis

We used MS Excel© macro *GENALEX* 3.6 (Peakall & Smouse, 2006) to calculate standard descriptive statistics of genetic diversity of burrowing owls at each of our study locations, including observed heterozygosity, expected heterozygosity, and fixation index *F*. We also used program *ARLEQUIN 3.1.1* (Excoffier, 2006) to estimate the Weir and Cockerham's *F_ST_* (*θ*, Weir & Cockerham, 1984) for all populations.

We computed actual differentiation *D* (measure of differentiation between populations independent of gene diversity) (Jost, 2008) to test our prediction that gene flow between declining migratory populations in the north and populations in southern agricultural areas would disrupt an otherwise apparent isolation‐by‐distance relationship. We used software *SMOGD* (Crawford, 2010)to compute actual differentiation *D*. We used *D* as our measure of population‐pairwise genetic differentiation because *F_ST_* does not adequately measure genetic differentiation when within‐population allelic diversity is high (Jost, 2008). *D* ranges from 0 to 1, corresponding to complete similarity to complete differentiation. We performed a Mantel test (Mantel, 1967) to test our assumption of the existence of an isolation‐by‐distance pattern (i.e., that the genetic differentiation between 2 populations is positively correlated to the geographic distance that separates those populations). If our hypothesis is true, pairwise comparisons between northern locations and southern agricultural locations will fall below the predicted Mantel regression line in the scatterplot of genetic versus geographic distances.

We performed an Analysis of Molecular Variance (*AMOVA;* Weir & Cockerham, 1984) using *ARLEQUIN* 3.1.1 to test our prediction that declining migratory populations in the north and populations in agricultural areas in the south, pooled together, would be genetically differentiated from the remainder of the breeding populations within the species' range (pooled together). *AMOVA* is analogous to a nested Analysis of Variance and uses a permutational approach to test the statistical significance of any given classification of study locations in explaining the overall genotypic variation. We performed 2 *AMOVA*s, one based on allele sizes (*R_ST_*) and the other based on the number of different alleles (*F_ST_*) (Michalakis & Excoffier, 1996). The former measure assumes the stepwise mutation model (Ohta & Kimura, 1973), which is appropriate for microsatellite loci. We used the *AMOVAs* to test for evidence of 2 distinct genetic groups: Group 1 with southern agricultural locations (CAG, CAB, CUL, FUE, HER, MEX, and YAQ) together with northern locations (ALB, SAK, and GRL), and Group 2 including all other locations. Our large sample size (1,560 individuals) may confer enough statistical power to reject the null hypothesis for any grouping of study locations. To explore this possibility, we conducted 7 additional *AMOVAs* using 2‐group classifications by replacing northern study locations (ALB, SAK, and GRL) from Group 1 with other study locations and moving them to Group 2.

We conducted an assignment test as implemented by the program *STRUCTURE* (Hubisz, Falush, Stephens, & Pritchard, 2009; Pritchard, Stephens, & Donnelly, 2000) to test our prediction that southern agricultural study locations will have more individual owls with probabilities of membership similar to those found in individuals from declining populations in the north compared to the nonagricultural study locations in the southern part of the species range. *STRUCTURE* 2.3.3 implements an algorithm suited to infer weak population structure (Hubisz et al., 2009). *STRUCTURE* estimates the posterior probability of the data (L(*K*) = Prob[*Data* |*K*]) given existence of *K* burrowing owl populations under Hardy–Weinberg equilibrium and estimates the posterior probability of membership of each individual owl to each of the *K* populations. We used study locations as prior information to assist the inference of population structure (Hubisz et al., 2009). We performed 10 runs for each *K* = 1, 2, … 10. Each run consisted of a burn‐in period of 50,000 Markov Chain Monte Carlo repetitions followed by 50,000 repetitions to sample from the posterior distribution of *K*. We estimated L(*K*) for each *K* from correlated allele frequencies and an admixture model. This approach is superior at detecting subtle genetic structure when population differentiation is low compared to the use of uncorrelated allele frequencies and a nonadmixture model (Falush, Stephens, & Pritchard, 2003). We used the outputs of the web‐based platform *STRUCTURE HARVESTER* 0.56.3 (http://taylor0.biology.ucla.edu/structureHarvester/) to assess the number of inferred populations. *STRUCTURE HARVESTER* estimates the statistic Δ*K* at each value of *K;* we used Δ*K* because it performs better in detecting population genetic structure than L(*K*) (Evanno, Regnaut, & Goudet, 2005). Therefore, actual number of populations is revealed by the value of *K* with the highest value of Δ*K*. We used program *CLUMPP* (Jakobsson & Rosenberg, 2007) to calculate the posterior probabilities of membership of each individual owl to each of the *K* populations from our multiple runs in *STRUCTURE*.

## 3 RESULTS

Burrowing owls exhibited high levels of genetic diversity (Table 2) with relatively low variation among study locations. Per‐locus average number of effective alleles (range 5.70–7.82), expected heterozygosity (range 0.78–0.84), observed heterozygosity (range 0.78–0.87), number of private alleles (alleles present at only 1 population, range 0.00–0.50), and fixation index (range −0.06–0.04) were similar among the 36 study locations (Table 2) in spite of the relatively large inter‐location variation in sample size (range 15–73; Table 1), and per‐locus average number of alleles (range 9.40–15.70; Table 2). We detected the possible occurrence of null alleles for locus ATCU13 at 2 study locations (BUC and CUL), for locus ATCU20 at 2 study locations (LAG and SAK), for locus ATCU39 at 1 study location (NTS), and for locus ATCU45 at 1 study location (MEX).

**Table 2:** Mean number of alleles (*N_a_*), number of effective alleles (*N_e_*), number of private alleles (*N_p_*), observed heterozygosity (*H_O_*), expected heterozygosity (*H_E_*), and fixation index (*F*) averaged across all 10 loci for each of 36 study locations of breeding burrowing owls in North America

Population	*N_a_*	*N_e_*	*N_p_*	*H_O_*	*H_E_*	*F*
ALB^†^	13.40 ± 1.72	7.24 ± 1.09	0.20 ± 0.13	0.83 ± 0.02	0.84 ± 0.02	0.00 ± 0.02
SAK^†^	15.70 ± 2.09	7.64 ± 1.38	0.20 ± 0.13	0.84 ± 0.02	0.84 ± 0.02	0.00 ± 0.03
GRL^†^	11.10 ± 1.29	6.98 ± 1.02	0.00 ± 0.00	0.85±0.03	0.83 ± 0.03	−0.03 ± 0.03
MOS	14.20 ± 1.36	7.37 ± 1.15	0.50 ± 0.40	0.84 ± 0.03	0.84 ± 0.02	0.00 ± 0.01
TCI	13.10 ± 1.68	7.13 ± 0.93	0.00 ± 0.00	0.86 ± 0.03	0.84 ± 0.02	−0.02 ± 0.03
BUF	14.20 ± 1.76	7.09 ± 1.33	0.20 ± 0.13	0.84 ± 0.02	0.83 ± 0.02	−0.01 ± 0.01
MNH	14.50 ± 1.90	7.36 ± 1.29	0.00 ± 0.00	0.84 ± 0.03	0.83 ± 0.02	−0.01 ± 0.01
PAW	14.20 ± 1.65	7.82 ± 1.37	0.00 ± 0.00	0.84 ± 0.02	0.84 ± 0.02	0.00 ± 0.02
DUG	11.70±1.14	6.66 ± 0.87	0.00 ± 0.00	0.86 ± 0.02	0.83 ± 0.02	−0.04 ± 0.02
BUC	12.00 ± 1.62	7.04 ± 1.12	0.00 ± 0.00	0.82 ± 0.04	0.82 ± 0.03	0.01 ± 0.03
CAR	10.40 ± 0.79	5.77 ± 0.65	0.00 ± 0.00	0.86 ± 0.03	0.81 ± 0.02	−0.06 ± 0.03
DIX	10.00 ± 1.03	6.17 ± 0.72	0.00 ± 0.00	0.86 ± 0.03	0.82 ± 0.02	−0.04 ± 0.04
COM	13.40 ± 1.76	7.42 ± 1.15	0.00 ± 0.00	0.87 ± 0.03	0.84 ± 0.02	−0.04 ± 0.02
NTS	11.30 ± 1.24	6.95 ± 0.70	0.00 ± 0.00	0.81 ± 0.03	0.84 ± 0.02	0.04 ± 0.03
LEM	12.60 ± 1.50	6.82 ± 1.27	0.10 ± 0.10	0.83 ± 0.03	0.82 ± 0.02	0.00 ± 0.02
KIB	11.90 ± 1.34	6.80 ± 1.05	0.00 ± 0.00	0.80 ± 0.03	0.82 ± 0.03	0.03 ± 0.02
NEL	12.50 ± 1.34	6.15 ± 0.57	0.00 ± 0.00	0.83 ± 0.02	0.82 ± 0.02	−0.01 ± 0.01
TXP	9.50 ± 1.26	5.95 ± 0.93	0.00 ± 0.00	0.78 ± 0.05	0.78 ± 0.04	0.00 ± 0.04
KIR	15.00 ± 1.56	7.34 ± 1.11	0.30 ± 0.30	0.84 ± 0.02	0.84 ± 0.02	0.00 ± 0.02
EDW	12.30 ± 1.10	6.58 ± 0.95	0.10 ± 0.10	0.83 ± 0.02	0.83 ± 0.02	0.00 ± 0.02
HOL	10.70 ± 1.18	6.81 ± 1.03	0.00 ± 0.00	0.86 ± 0.04	0.83 ± 0.02	−0.04 ± 0.03
WSM	11.70 ± 1.09	7.11 ± 0.98	0.10 ± 0.10	0.85 ± 0.03	0.84 ± 0.02	−0.02 ± 0.02
TUC	9.40 ± 1.13	5.70 ± 0.76	0.00 ± 0.00	0.78 ± 0.04	0.79 ± 0.03	0.01 ± 0.03
JAN	14.10 ± 1.63	7.22 ± 1.21	0.20 ± 0.13	0.86 ± 0.02	0.83 ± 0.02	−0.03 ± 0.01
CHI	12.30 ± 1.29	6.66 ± 0.92	0.00 ± 0.00	0.82 ± 0.03	0.83 ± 0.02	0.01 ± 0.02
DEL	11.20 ± 1.11	6.60 ± 0.87	0.30 ± 0.21	0.83 ± 0.03	0.82 ± 0.02	−0.01 ± 0.03
LAG	14.20 ± 1.73	7.42 ± 1.19	0.20 ± 0.13	0.83 ± 0.03	0.84 ± 0.02	0.01 ± 0.03
BCS	11.00 ± 1.26	6.36 ± 0.96	0.20 ± 0.13	0.81 ± 0.04	0.82 ± 0.02	0.02 ± 0.03
GAL	13.30 ± 1.56	7.25 ± 1.17	0.00 ± 0.00	0.84 ± 0.02	0.84 ± 0.02	−0.01 ± 0.02
CAG*	14.70 ± 1.76	6.88 ± 1.07	0.00 ± 0.00	0.84 ± 0.02	0.83 ± 0.02	−0.01 ± 0.01
MEX*	13.70 ± 1.67	7.20 ± 1.22	0.10 ± 0.10	0.83 ± 0.03	0.83 ± 0.02	0.00 ± 0.02
CAB*	10.80 ± 1.27	6.70 ± 0.78	0.00 ± 0.00	0.84 ± 0.03	0.83 ± 0.02	−0.01 ± 0.02
HER*	13.40 ± 1.30	6.92 ± 1.09	0.00 ± 0.00	0.83 ± 0.02	0.83 ± 0.02	0.00 ± 0.02
YAQ*	13.50 ± 1.55	6.79 ± 0.96	0.20 ± 0.20	0.82 ± 0.02	0.83 ± 0.02	0.01 ± 0.01
FUE*	13.50 ± 1.68	6.85 ± 0.98	0.10 ± 0.10	0.83 ± 0.03	0.82 ± 0.03	0.00 ± 0.02
CUL*	13.00 ± 1.81	7.29 ± 1.28	0.00 ± 0.00	0.81 ± 0.04	0.82 ± 0.04	0.02 ± 0.01

Population acronyms are shown in Table 1. Study location acronyms with (*) and (†) denote southern agricultural populations and northern declining migratory populations, respectively. Different shades denote population membership based on >50% of the posterior probability of membership from the program STRUCTURE (see Figure 3).

**Figure 2:**
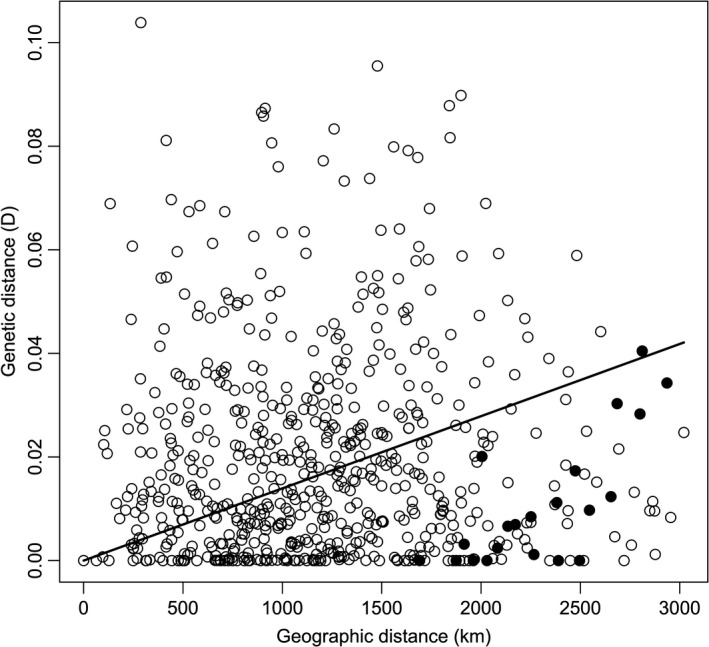
Scatterplot of actual differentiation *D* versus geographic distances for all pairwise comparisons (*n* = 630) among our 36 burrowing owl study locations across North America. Black dots indicate pairwise comparisons between northern study locations and southern agricultural locations, whereas empty dots indicate pairwise comparisons among the remainder of the study locations. Mantel correlation between geographic and genetic distance was not statistically significant (95% CI from −0.05 to 0.08), but the relationship was positive as expected (i.e., more distant populations tended to be less similar)

Burrowing owls had low levels of genetic differentiation among study locations as shown by relatively low overall *F_ST_* (*θ* = 0.008) and low pairwise *F_ST_* statistics (${\bar{F}}_{\mathrm{ST}}$ = 0.0113 ± 0.0002, *n* = 630). Low levels of genetic differentiation were also evident in our estimates of actual differentiation *D*, ranging from 0.00 to 0.11. In this regard, we found a subtle, positive relationship between genetic distance and geographic distance among our study locations (Figure 2). The Mantel test was not significant (*r* = 0.015, *p* = .43 based on 1,000 permutations) but the slope was positive (as would be predicted by reduced dispersal frequency among populations further and further apart). Moreover, all but one of the pairwise comparisons of genetic and geographic distances among northern study locations and southern agricultural locations fall below the Mantel regression line (Figure 2), an important prediction of the migration‐mediated breeding dispersal hypothesis.

Low levels of genetic differentiation among populations were also highlighted by our *AMOVA*s based on the *R_ST_* and *F_ST_* statistics. Genetic variation within study locations explained 99% of the total genetic variation, whereas between‐study locations and between two‐group classifications of study locations explained the remaining 1%. Despite the low levels of genetic differentiation described above, our *AMOVA* based on the *F_ST_* statistic also provided support to the migration‐driven breeding dispersal hypothesis. Both a standard *AMOVA* and a weight‐averaged *AMOVA* over all loci provided suggestive evidence that northern study locations (ALB, SAK, and GRL) and southern agricultural study locations (CAG, CAB, CUL, FUE, HER, MEX, and YAQ) together are genetically differentiated from the rest of the study locations (*p* = .03 and *p* = .01, respectively) although this result did not hold true for the 2 *AMOVA*s based on *R_ST_* (*p = *.38 and *p* = .34, respectively). In addition, only 1 of the 7 additional *AMOVA*s based on *F_ST_* was significant for both the standard *AMOVA* and the weight‐averaged *AMOVA* over all loci (Table 3), which is precisely the *AMOVA* that included the nearest 3 study locations (CHI, JAN, and TUC) within Group 1.

**Table 3:** Statistical significance (*p*‐values) of Analyses of Molecular Variance (*AMOVA*) based on the *F_ST_* statistics for each of 8 two‐group classifications of 36 burrowing owl breeding locations in North America

Study sites in Group 1	*p*‐value	
	Standard	Weighted averaged over all loci
**ALB, GRL, SAK**	**.028**	**0.012**
MNH, MOS, TCY	.240	0.218
BUF, CAR, PAW	.131	0.117
EDW, NEL, NTS	.220	0.238
COM, KIB, KIR	.184	0.174
DEL, GAL, LAG	.060	**0.046**
DIX, LEM	.329	0.328
**CHI, JAN, TUC**	**.027**	**0.008**

Note

Group 1 includes the southern agricultural study sites (CAB, CAG, CUL, FUE, HER, MEX, and YAQ) and the study sites listed in the table below. Group 2 includes the remainder of the study sites. Acronyms are listed in Table 1. Bold‐face values denote significant comparisons (*p* ≤ .05).

**Figure 3:**
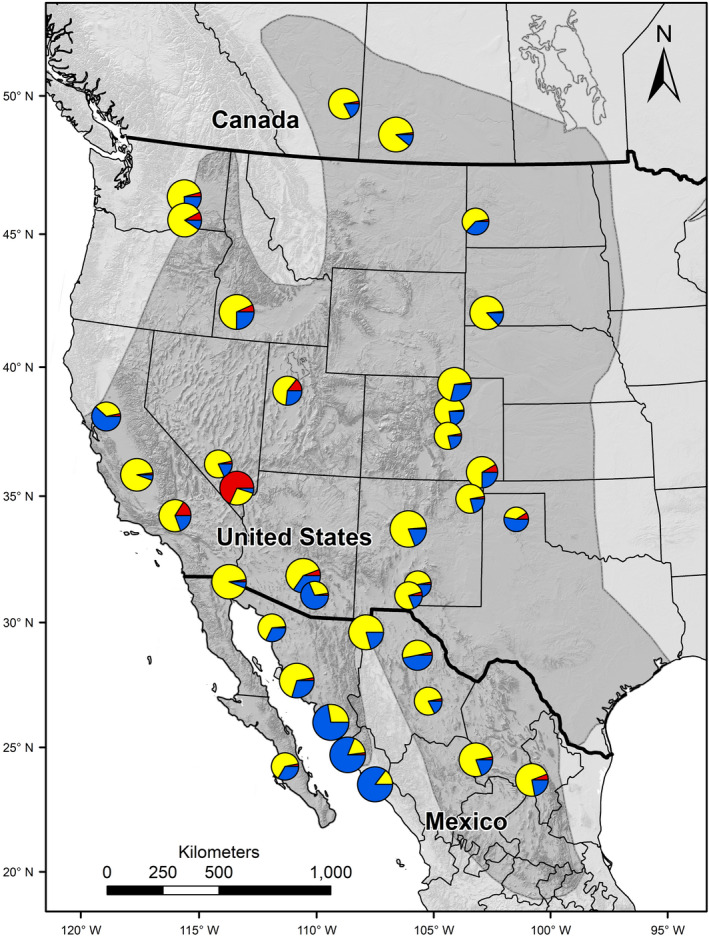
Geographic variation among burrowing owl study locations in the posterior probability of membership to each of the 3 populations inferred by program *STRUCTURE*. Pie chart sizes are proportional to the number of individuals genotyped at each study location. The gray area denotes the breeding distribution of the burrowing owl (after Poulin et al., 2020)

*STRUCTURE* revealed a genetic structure consisting of 3 populations in the burrowing owl in spite of the low levels of genetic differentiation among study locations shown by *F_ST_* and *D* statistics. Mean log‐likelihood of the observed genotypic data and Δ*K* was highest at *K* = 3 (indicating 3 distinct populations, see Figure S1). The posterior probabilities of membership of each of our 1,560 individual owls assigned to these putative populations (see Figure S2) had a noticeable geographic pattern (Figure 3). Almost all burrowing owls in southern agricultural study locations in southern Sonora (YAQ) and Sinaloa (FUE and CUL) had a higher probability of membership to one inferred population (Sinaloan population). This genetic structure was corroborated by a standard *AMOVA* (based on the *F_ST_*) which differentiates this Sinaloan population (CUL, FUE, and YAQ) from the rest of the study locations (*p* = .005). This Sinaloan fingerprint is relatively common within nearby populations in Sonora, southern Arizona, and as far as Chihuahua (CHI), northern Texas (TXP), and the Central Valley of California (DIX) (blue color in pie charts in Figure 3). Similarly, burrowing owls from Nellis Air Force Base in southern Nevada (NEL) were distinctive (Mojave population), and their fingerprint also appears in burrowing owl populations in the western portion of the breeding range in Washington, California, and Utah (red color in pie charts in Figure 3). Finally, the great majority of the individuals in the remainder of the study locations, including northern study locations, had the fingerprint of a third inferred population (North American population, yellow color in pie charts in Figure 3) where northern study locations and the northern half of the southern agricultural study locations (HER, CAB, MEX, and CAG) are included. Hence, results of the STRUCTURE analysis did not support our hypothesis but it did suggest some subtle population structure that is interesting. Individual owls from 4 southern agricultural study locations (CAG, MEX, CAB, and HER) had similar probabilities of membership to those found in owls from northern locations but also similar to those found in owls from nonagricultural study locations in the southern part of the range (e.g., JAN and GAL). In addition, probabilities of membership were remarkably different in owls from the 3 southernmost agricultural locations (CUL, FUE, and YAQ), compared to those found in owls from northern locations (ALB, SAK, and GRL).

## 4 DISCUSSION

Burrowing owl populations in North America have low levels of differentiation as shown by *F_ST_* and *D* statistics and, in that regard, our results corroborate a previous study that also reported low levels of genetic differentiation for the western burrowing owl (Korfanta, McDonald, & Glenn, 2005

). Korfanta et al. (2005) estimated *F_ST_* = 0.01 (95% CI: 0.007–0.02) and concluded that burrowing owl populations were practically panmictic. Our estimate of *F_ST_* = 0.008 is slightly lower but still within the 95% confidence interval of their *F_ST_* estimate. Our study was more comprehensive than Korfanta et al. (2005) because it represents a 10‐fold increase in the number of individuals (155 vs. 1,560) and fourfold increase in the number of study locations (9 vs. 36), and we included populations in Mexico and Canada. Our study also represents a 43% increase in the number of microsatellite loci used (7 vs. 10). In addition, markers used in this study were more variable, with an average of 11.6 alleles per locus (range 5–25, Macías‐Duarte et al., 2010) versus 8.3 alleles per locus (range 3–19, Korfanta et al., 2005). Therefore, burrowing owls clearly have low genetic differentiation among populations that extends throughout the entire breeding range in North America (including populations in Canada and Mexico), which is not surprising for a migratory bird. However, a major assumption for our 3 predictions is that burrowing owl populations had at least some subtle genetic structure before the development of the agricultural valleys in southwestern United States and northwestern Mexico. This low genetic population differentiation throughout the burrowing owl breeding range hindered our ability to rigorously test the migration‐driven breeding dispersal hypothesis. Despite the challenges associated with the minimal genetic structure, we did detect some tentative support for the migration‐driven breeding dispersal hypothesis.

The 10 DNA microsatellite loci we used may have been too few to detect subtle genetic structure using the *F_ST_* index. Hence, the use of our genetic markers to detect past and current patterns of breeding dispersal is imperfect. We used numerous analytical methods and algorithms that made use of the individuals' genotypic data (e.g., program *STRUCTURE*) to unveil subtle patterns of genetic differentiation. Estimates of Δ*K* revealed a subtle genetic structure and identified 3 populations. However, Δ*K* cannot be computed for *K* = 1 (Evanno et al., 2005) and therefore the scenario of 1 single population in Hardy–Weinberg equilibrium is still possible given our low values for *F_ST_* and *D*. However, consistent geographic patterns in probabilities of membership suggest that our results have validity. *STRUCTURE* is a spatially blind analysis because geographic coordinates are not an input in the analysis. Therefore, the fact that the 3 southernmost agricultural populations (CUL, FUE, and YAQ in northwestern Mexico) (Figure 3) all had higher probability of membership to a single population suggests that the inferred population structure is real. The proportion of migratory individuals in burrowing owl populations across the species' breeding range is positively correlated to both latitude and elevation, but also subject to local environmental control (Ogonowski & Conway, 2009). Therefore, burrowing owl populations in the coastal, subtropical agricultural areas of southern Sonora and Sinaloa are likely formed mostly or completely by nonmigratory birds. Accordingly, the observed genetic structure suggests that irrigated agriculture in Sonora and Sinaloa has influenced population dynamics of burrowing owls and has created populations that are subtlety distinct from the rest of the populations within the breeding range, distinct even from the neighboring agricultural populations in central Sonora and those in the Colorado River delta. Although *STRUCTURE* did not support a direct link between southern agricultural locations and the northern‐most locations, our *AMOVA* did provide evidence of such a link. Our *AMOVA* based on *F_ST_* provided support for the predicted pattern of breeding dispersal from northern locations to southern agricultural locations, differentiating this group from other burrowing owl locations. In contrast, our *AMOVA* based on allele sizes (*R_ST_*) did not provide support of the hypothesis. However, measures of allele size have been criticized for having large sampling errors and low efficiency in reconstructing simulated phylogenies (Takezaki & Nei, 1996). In addition, the lack of statistical significance in 6 of the 7 additional *AMOVA*s (Table 3) suggests that the genetic connectivity inferred between southern agricultural locations and northern locations is not an artifact of our large sample size (1,560 individuals and 10 loci). In fact, the only other significant *AMOVA* included southern agricultural locations and neighboring Tucson (TUC), Janos (JAN), and Ahumada and Coyame (CHI) locations in Group 1, which makes sense because of regional gene flow.

The validity of the genetic structure found in this study is corroborated by the known current patterns of migratory connectivity in the burrowing owl. Recent satellite telemetry data (C. J. Conway, unpublished data) revealed that burrowing owls breeding in Great Plains winter through the Mexican Highlands and the eastern coastal plains of the Gulf of California, whereas burrowing owls breeding west of the Rocky Mountains winter in California and the Baja California Peninsula. Accordingly, the distinctive Mojave population centered in southern Nevada (red sections in Figure 3) suggests more limited gene flow from burrowing owl breeding populations lying on the Great Plains migratory pathway. A latitudinal breeding dispersal via migratory behavior predicts this genetic structure. However, the explanation for the stronger genetic signal for the NEL location is not evident. Nevertheless, the latitudinal genetic structure across burrowing owl populations found here may have preceded the agricultural land development in northwestern Mexico, and this structure may have been retained after changes in land use. Alternatively, northern populations may have been derived from the southern populations as a postglacial range expansion.

In summary, our study provides some intriguing evidence that declines near the northern edge of the breeding range of burrowing owls may be at least partially caused by migration‐driven dispersal to newly created irrigated agricultural areas in northwestern Mexico. However, low levels of genetic differentiation among populations hindered the resolution of our analysis. Stable isotopes and more extensive banding and resighting efforts in Canada and the irrigated agricultural areas are two approaches that would further test the migration‐driven breeding dispersal hypothesis. Our results are part of a growing body of literature that document the influence of the land‐use mosaic on the distribution and movement of animals, and such shifts can produce location‐specific population dynamics leading to subtle genetic structure even in the absence of dispersal barriers or isolation by distance. The long‐term conservation value of agroecosystems in Sonora and Sinaloa should be evaluated because these newly created ecosystems support dense breeding populations of burrowing owls—likely higher than any other areas in North America.

Despite the limited genetic differentiation among populations, genetic diversity in DNA microsatellite loci among our sampling locations was higher than that found in other owl species of wide distribution, and in other owl species of conservation concern. Average expected heterozygosity per locus across study locations ranged from 0.77 to 0.86 for burrowing owls (36 locations, this study), from 0.54 to 0.62 in the ferruginous pygmy‐owl *Glaucidium brasilianum* (8 locations, Proudfoot, Honeycutt, Slack, & Ingraldi, 2006), from 0.48 to 0.56 in the boreal owl *Aegolius funereus* (6 locations, Koopman, Hayward, & McDonald, 2007), from 0.47 to 0.63 in great gray owls *Strix nebulosa* (5 locations, Hull et al., 2010), and from 0.72 to 0.77 in the spotted owl *Strix occidentalis* (6 locations, Funk, Forsman, Johnson, Mullins, & Haig, 2010). Similarly, low genetic differentiation also been documented in the boreal owl (*F_ST_* = 0.004 using microsatellite loci; Koopman et al., 2007), and the flammulated owl *Otus flammeolus* (*F_ST_* < 0.04 using DNA fingerprinting; Arsenault, Stacey, & Hoelzer, 2005), as well as the endangered northern spotted owl *Strix occidentalis caurina* (*F_ST_* = 0.024 using microsatellite loci; Funk et al., 2010). Substantial genetic structure has been documented for the great gray owl (*F_ST_* < 0.17 from microsatellite loci; Hull et al., 2010). Low levels of genetic differentiation among populations of burrowing owls are highly relevant for burrowing owl conservation and restoration programs everywhere in North America. Low genetic differentiation among our 36 study locations from Canada to central Mexico provides further evidence that burrowing owls are a large panmictic population across the species' breeding range. Reintroduction programs may be able to use individuals from populations throughout western North America without substantially compromising genetic variation for local adaptation. Low genetic differentiation, presumably caused by continent‐wide breeding dispersal, also means that population trends in a given location may be caused by changes in demographic processes (e.g., fecundity, mortality, and emigration) in other portions of the species' range. Therefore, population declines in the northern edge of the species' breeding distribution may reflect either declines in immigration from more interior populations, low local recruitment, or both.

## CONFLICT OF INTEREST

None declared.

## AUTHOR CONTRIBUTIONS

**Alberto Macias‐Duarte:** Conceptualization (supporting); data curation (lead); formal analysis (lead); investigation (equal); methodology (equal); writing–original draft (lead); writing–review and editing (equal). **Courtney J. Conway:** Conceptualization (lead); funding acquisition (lead); investigation (supporting); resources (equal); writing–original draft (equal); writing–review and editing (equal). **Melanie Culver:** Formal analysis (equal); investigation (equal); methodology (equal); resources (lead); supervision (equal); writing–original draft (equal); writing–review and editing (equal).

## Supporting information

Fig S1

Fig S2

## Data Availability

Data from this manuscript, including DNA microsatellite genotypes and sampling locations, were archived in the publicly accessible repository Dryad (https://doi.org/10.5061/dryad.vdncjsxrx).
