# Supplementary figures and images for: Agriculture creates subtle genetic structure among migratory and nonmigratory populations of burrowing owls throughout North America
Source: Ecol Evol. 2020 Sep 17;10(19):10697–708. doi: 10.1002/ece3.6725 (PMC7548177; doi:10.1002/ece3.6725)

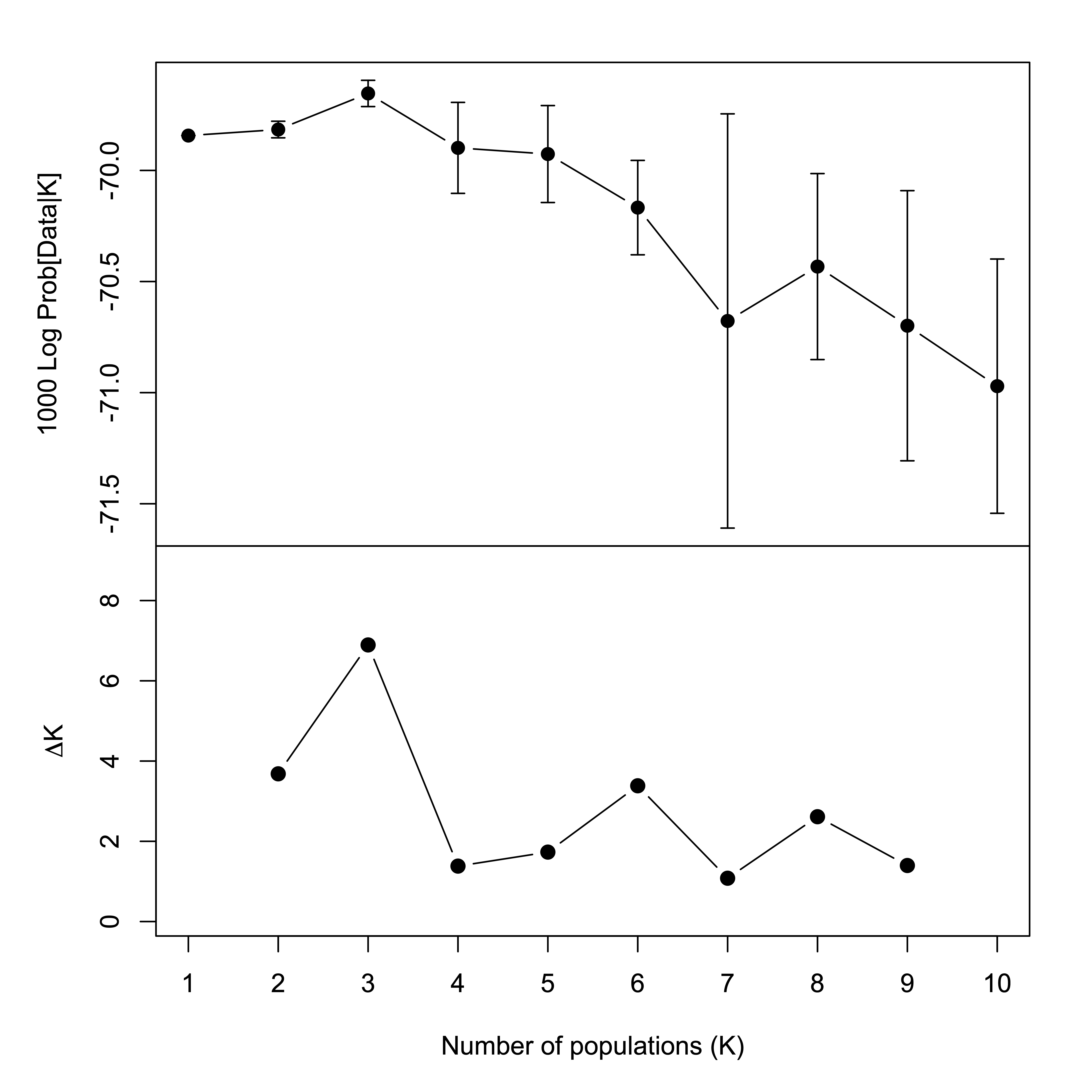

Supplement: Supplementary file 1 — Fig S1 [file ECE3-10-10697-s001.jpg]

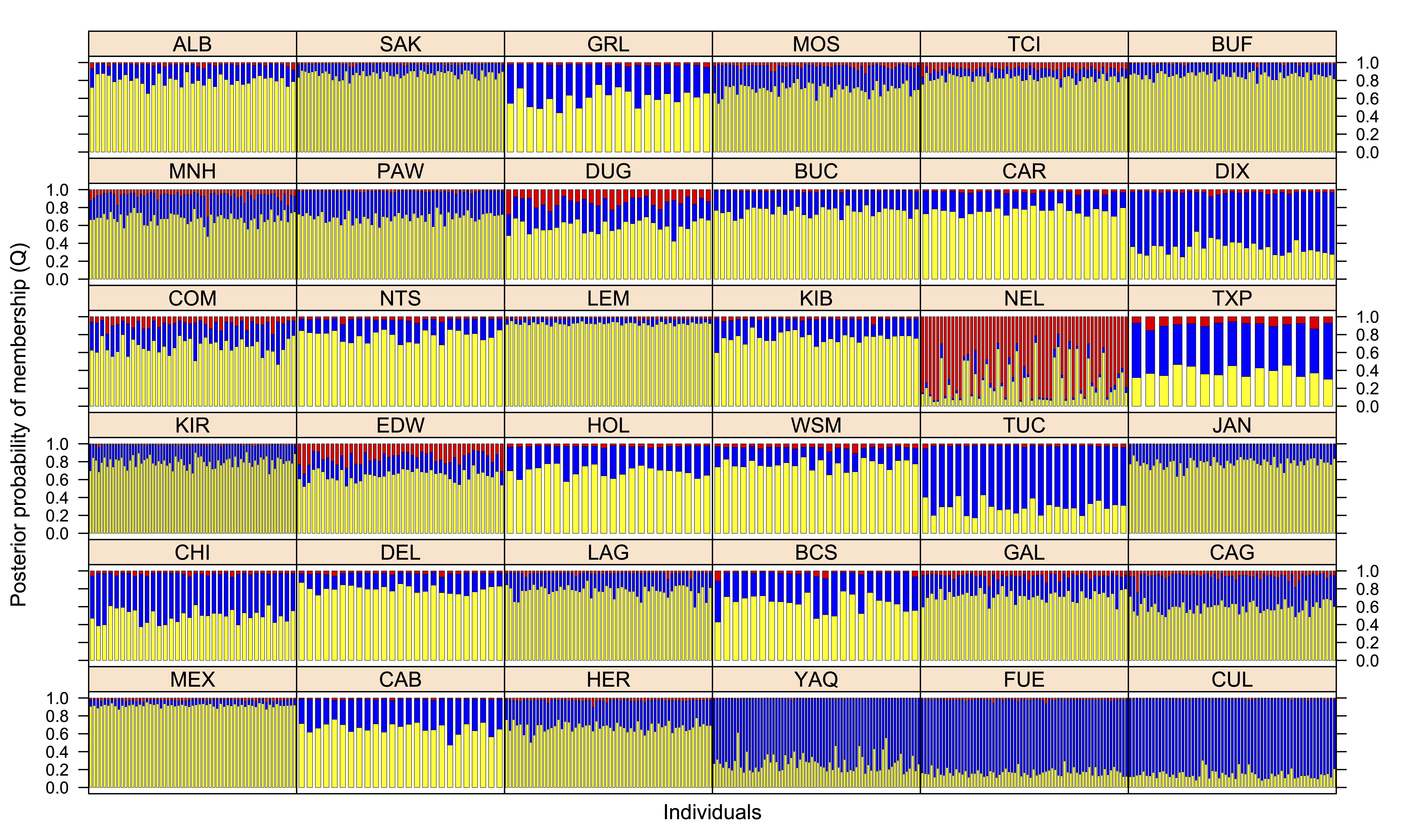

Supplement: Supplementary file 2 — Fig S2 [file ECE3-10-10697-s002.jpg]
